# Floss abrasion: a cautionary tale

**DOI:** 10.1007/s40368-020-00562-6

**Published:** 2020-09-18

**Authors:** J. Cooper, D. Al-Jassim, S. Barry

**Affiliations:** University Dental Hospital of Manchester, Higher Cambridge Street, Manchester, M15 6FH England

**Keywords:** Hall Technique, Preformed Metal Crown, Orthodontic Separator, Abrasion

## Abstract

**Background:**

The Hall technique for placement of preformed metal crowns is widely used in the UK for the management of decayed primary molar teeth. The creation of space is achieved by the placement of orthodontic separators adjacent to the tooth requiring restoration. Highlighting the first reported case of an abrasion caused by dental floss, this communication describes the clinical findings of an 8-year-old patient following placement of orthodontic separators.

**Case report:**

An 8-year old boy attended the University Dental Hospital of Manchester for placement of orthodontic separators prior to restoration URE, ULE, LLE, and LRE with preformed metal crowns using the hall technique. The following week he presented with a 2cm abrasion to his right cheek, which had been caused by dental floss used in placement of the orthodontic separators.

**Follow up:**

Conservative advice was given and the lesion had resolved completely at a 2-week review.

**Conclusion:**

This case is a timely reminder of the importance of adequate lip retraction and soft tissue management during placement of orthodontic separators.

We wish to highlight a case of an iatrogenic facial abrasion caused by dental floss during the placement of an orthodontic separator. An 8-year-old boy attended the undergraduate student clinic for placement of separators, prior to the placement of preformed metal crowns using the Hall technique URE, ULE, LLE and LRE. The procedure was uneventful. However, the following week the patient presented with a 2 cm abrasion to his right cheek which was healing (Fig. 1). His mother noted this had been caused by dental floss used in the placement of the separators the previous week. To our knowledge this is the first reported case of abrasion by dental floss reported in the literature. .

**Fig. 1:**
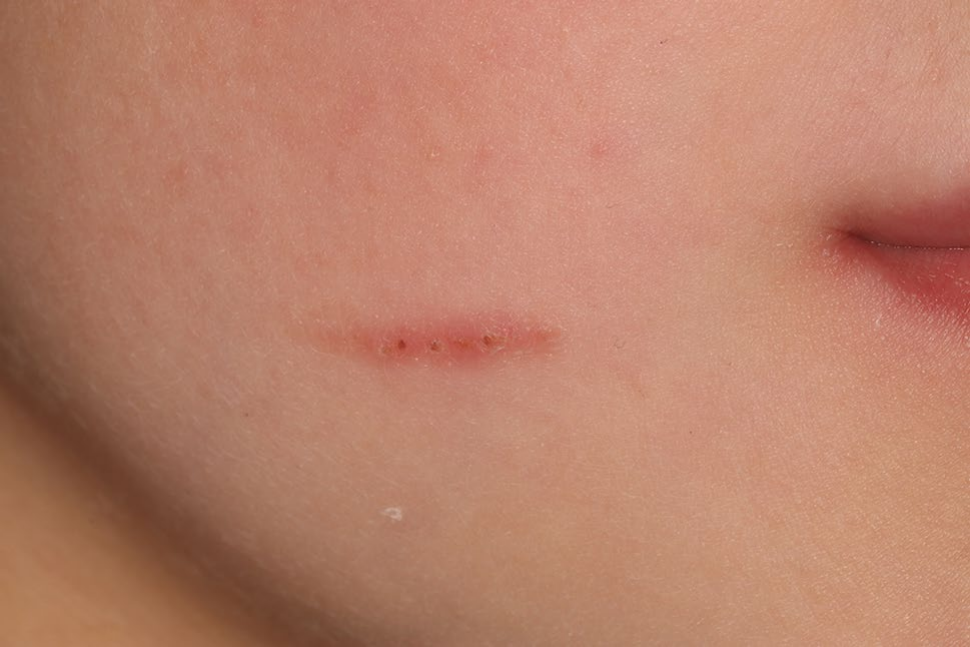
A clinical photograph showing the abrasion lesion on the right cheek

Treatment of an abrasion involves cleaning the area, application of petroleum jelly and careful follow-up to assess for infection or scarring. In this case, the lesion had resolved completely at a 2-week review.

Preformed metal crowns are the gold standard restoration for extensively decayed primary molar teeth (Kindelan et al. 2008). The Hall technique is widely used in the UK as it is acceptable to most children and provides effective treatment without the use of local anaesthetic or need for tooth preparation (Innes et al. 2015). Classified as a non-aerosol generating procedure, the use of this technique is advocated during the COVID-19 pandemic for management of caries in the primary dentition (Al-Halabi et al. 2020). The use of the Hall technique is a universally taught competency during undergraduate dental training in the UK. The creation of space is usually achieved by the placement of elastic separators mesial and distal to the tooth in question. Although orthodontic separator placement pliers can be used, application with dental floss is also a widely used approach. Whilst placing the separators clinically, students may focus on the task at hand and unknowingly lack perception of surrounding soft tissues. This case is a timely reminder of the importance of adequate lip retraction and soft-tissue management.

## References

[CR1] Al-Halabi M, Salami A, Alnuaimi E (2020). Assessment of paediatric dental guidelines and caries management alternatives in the post COVID-19 period. A critical review and clinical recommendations. Eur Arch Paediatr Dent.

[CR2] Innes N, Ricketts D, Chong L, Keightley A (2015). Preformed crowns for decayed primary molar teeth. Cochrane Database Syst Rev.

[CR3] Kindelan SA, Day P, Nichol R, Willmott N (2008). UK national clinical guidelines in paediatric dentistry: stainless steel preformed crowns for primary molars. Int J Paediatr Dent.

